# Are there differences in arthroscopic and histological features between traumatic and degenerative rotator cuff tears in elderly patients? A prospective dual-center analysis

**DOI:** 10.1186/s13018-022-03100-w

**Published:** 2022-04-07

**Authors:** Terufumi Shibata, Teruaki Izaki, Jun Nishio, Satoshi Miyake, Yasuhara Arashiro, Tomohiko Minamikawa, So Minokawa, Yozo Shibata, Takuaki Yamamoto

**Affiliations:** 1 Department of Orthopaedic Surgery, Fukuoka University Chikushi Hospital, 1-1-1 Zokumyoin, Chikushino, Fukuoka 818-8502 Japan; 2 Department of Orthopaedic Surgery, Fukuoka University Faculty of Medicine, 7-45-1 Nanakuma, Jonan-ku, Fukuoka, 814-0180 Japan

**Keywords:** Traumatic rotator cuff tears, Elderly, Repair tension, Coracoacromial ligament, Arthroscopy, Histology

## Abstract

**Background:**

Discriminating traumatic rotator cuff tears (RCTs) from degenerative RCTs is sometimes difficult in elderly patients because the prevalence of asymptomatic RCTs increases with age. Little intraoperative information is available on the characteristics of traumatic and degenerative RCTs in elderly patients. The purpose of this study was to compare the arthroscopic findings and histological changes of the coracoacromial ligament (CAL) between traumatic and degenerative RCTs in elderly patients.

**Methods:**

Forty-two shoulders of 42 patients aged ≥ 65 years underwent arthroscopic rotator cuff repair. Nineteen patients had traumatic full-thickness RCTs (Group T), and 23 had degenerative full-thickness RCTs (Group D). The quality of the rotator cuff tissue and the condition of the long head of the biceps were examined. The grade of CAL was evaluated both arthroscopically and histologically. The stiffness of the musculotendinous unit was calculated by measuring the force and displacement using a tensiometer. The arthroscopic and histological findings of the two groups were compared.

**Results:**

Although the mean tendon displacement was comparable, the stiffness was different between Group T and Group D (0.56 ± 0.31 and 1.09 ± 0.67 N/mm, respectively; *p* < 0.001). Both arthroscopic and histological analysis of the CAL showed that the degenerative changes in the CAL were milder in Group T than in Group D (*p* < 0.001 and *p* < 0.001, respectively). There was a moderate positive correlation between the arthroscopic findings of CAL degeneration and the histopathological changes in this ligament (r = 0.47, *p* = 0.002).

**Conclusions:**

Traumatic RCTs were characterized by preserved elasticity of the musculotendinous unit and milder CAL degeneration compared with degenerative RCTs even in elderly patients.

## Background

The incidence of rotator cuff tears (RCTs) increases with age, and the prevalence of asymptomatic RCTs is approximately 10.5% in patients aged ≥ 65 years [1]. Diagnosing acute traumatic RCTs by only examining the patient’s medical history may be difficult because preexisting asymptomatic RCTs can become symptomatic due to trauma (acute-on-chronic tear) [2]. In such patients, evaluating whether trauma is the cause of tendon rupture is important for making decisions in cases involving workers’ compensation or personal accident insurance [2, 3]. Early surgical intervention for traumatic RCTs is generally recommended to improve the functional outcome of the shoulder [4–7]. Studies of animal models have shown that the elasticity of the rotator cuff gradually disappears after tendon detachment, and this can lead to a decrease in failure strength [8, 9]. Traumatic rupture may occur in tendons with age-associated degeneration [10], but the elasticity of the musculotendinous unit is assumed to be preserved even in elderly patients with traumatic RCTs. High tension at the repair site has been considered one cause of retear [11–13]. If the musculotendinous unit is more elastic in traumatic than degenerative RCTs, the edge of the traumatic tear could more easily reach the footprint. This would be very informative for elderly patients to avoid excessive tension in the tendon, which provides an advantage for cuff healing because older age is associated with stiffness of the tendon [14, 15].

The cause of degenerative RCTs is believed to involve degenerative change of the tendon with age [16] and extrinsic factors such as subacromial impingement [17]. Although the correlation between RCTs and extrinsic factors is controversial, the subacromial impingement theory remains entrenched [18, 19]. Because the undersurface of the acromion and the coracoacromial ligament (CAL) are responsible for this mechanical impingement [17], we presume that the degeneration of the CAL is milder in traumatic than in degenerative RCTs.

To our knowledge, little intraoperative information is available on the characteristics of traumatic and degenerative RCTs in elderly patients. The purpose of this study was to compare the arthroscopic findings and histological changes of the CAL between traumatic and degenerative RCTs in elderly patients. The hypothesis was that preserved elasticity of the musculotendinous unit and mild CAL degeneration are specific features of traumatic RCTs in elderly patients.

## Methods

This prospective dual-center study involved elderly patients who underwent arthroscopic rotator cuff repair for traumatic or degenerative RCTs. From July 2018 to March 2020, all patients aged ≥ 65 years with full-thickness tears of the supraspinatus and/or infraspinatus tendon diagnosed by preoperative magnetic resonance imaging (MRI) were included in this study. Institutional review board approval was obtained from each institution. Informed consent was obtained from all patients before participation. Exclusion criteria were partial-thickness RCTs, displaced fractures around the shoulder girdle, a history of shoulder surgery, infections, degenerative or neuropathic arthritis, known contralateral shoulder RCTs, massive RCTs requiring margin convergence or medialization of the footprint, and severe fatty degeneration of the rotator cuff muscles (Goutallier classification grade ≥ 3) [20].

With reference to previous studies [2, 6, 21, 22], traumatic RCTs were defined as follows: sudden onset, unexpected traumatic event determined by a specific date and place, falling onto the outstretched arm from standing height or a traffic accident resulting in injury, no previous shoulder symptoms, and minimal fatty degeneration of the rotator cuff muscles (Goutallier classification grade ≤ 2) [20] as determined by one orthopedic surgeon during preoperative MRI at each institution [23]. Grade ≤ 2 fatty infiltration of the rotator cuff muscles is characteristic of acute RCTs [6, 9], while chronic shoulder pain or malfunction without trauma are the diagnostic criteria for degenerative RCTs [2, 5]. When patients with severe fatty degeneration were included, traumatic and degenerative RCTs could be easily differentiated only by MRI findings; therefore, only patients with minimal fatty degeneration of the rotator cuff muscles were included in our study.

All surgeries were performed by one of three senior shoulder surgeons. Each surgery was performed with the patient in the beach chair position under general anesthesia with interscalene block. Intraarticular lesions were evaluated through the standard posterior portal. When a subscapularis tendon tear was found, debridement or single-row repair was performed. The condition of the long head of the biceps (LHB) was examined. LHB tenotomy was performed for LHB lesions such as subluxation, dislocation, or a partial tear of ≥ 50%.

**Fig. 1:**
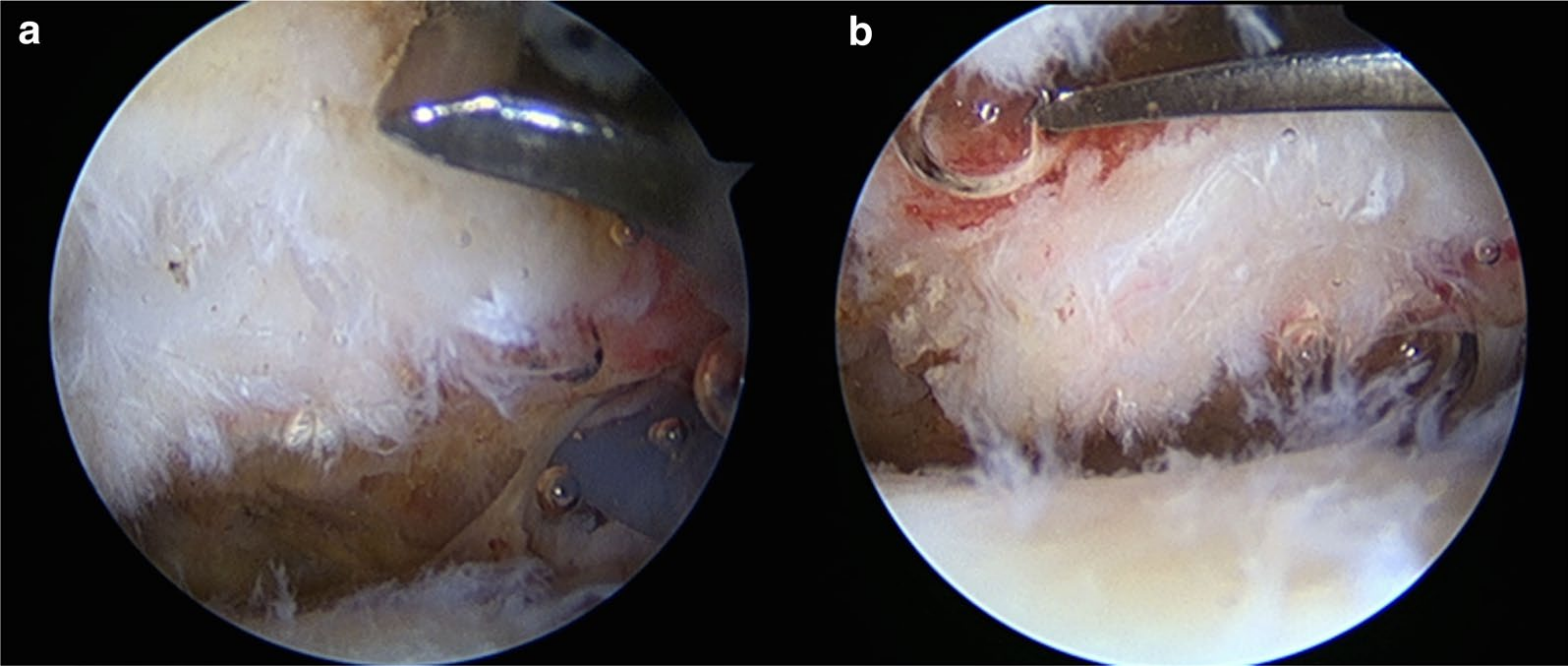
**a** Observation from the posterolateral portal of the right shoulder. Grade 2 coracoacromial ligament degeneration was confirmed. **b** The acromion and the coracoacromial ligaments were removed using a chisel

The arthroscope was then introduced into the subacromial space from the posterolateral portal. Bursal tissue was removed to clearly observe the undersurface of the acromion. The degeneration of the CAL attachments at the undersurface of the acromion was evaluated using the Copeland–Levy classification (Grade 0: normal appearance, Grade 1: minor scuffing, Grade 2: major scuffing, Grade 3: bare bones area) [24]. The CAL was cut immediately distal to its attachment to the acromion. A chisel with a width of 1 cm was introduced from the anterolateral portal. While observing from the posterolateral portal, the acromion and CAL were removed using the chisel (Fig. 1a, b).

**Fig. 2:**
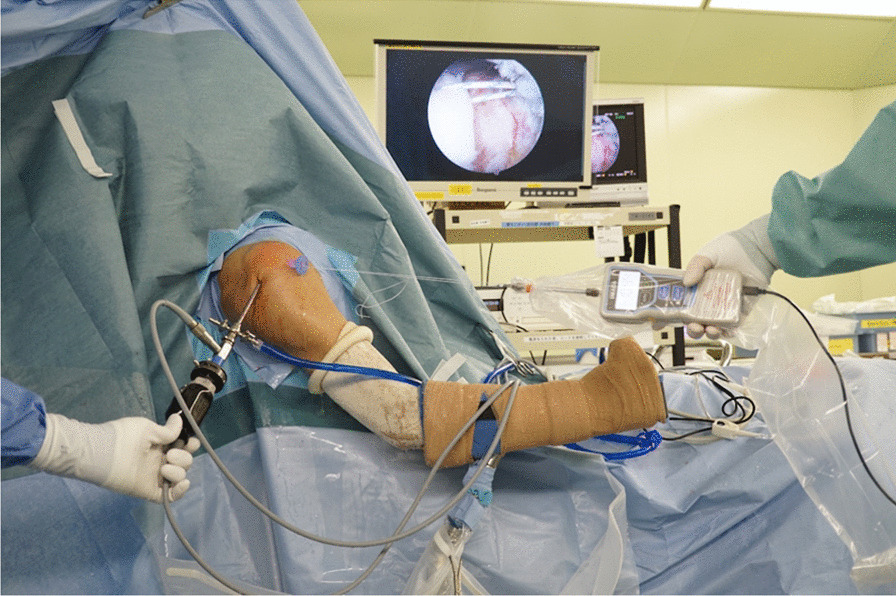
Measurement of the torn rotator cuff tension. A nonabsorbable suture that grasped the end of the tendon was pulled through the anterolateral portal and then tied to make a closed loop. This suture loop was connected to the sterile hook of a digital tensiometer. Tension was forced to the tensiometer until full coverage of the footprint was attained

The maximum length from anterior to posterior and from medial to lateral of the RCT was measured using a ruler. The tear size was also classified using the system reported by Cofield et al. [25]: small (< 1 cm), medium (1 to < 3 cm), large (3 to < 5 cm), or massive (≥ 5 cm). The following criteria of the rotator cuff tendon status described by Collin et al. [26] were used to evaluate soft tissue quality: good soft tissue quality was defined as satisfactory rotator cuff tissue with normal quality and thickness, moderate soft tissue quality was defined as a firm tendon with at least half its normal thickness, and poor soft tissue quality was defined as a soft or friable tendon with less than one-half its normal thickness. If tendon mobilization was insufficient in patients with larger tears, a superficial and deep adhesion was released from the rotator cuff and coracohumeral ligament. The torn edge of the tendon was grasped and translated medial to lateral or anterior to posterior to confirm the direction of the anatomical reduction [27]. Once the direction of tendon reduction was determined, a stitch was placed 1 cm from the end of the supraspinatus tendon using non-absorbable suture. The suture was pulled through the anterolateral portal and tied to make a closed loop. When this direction and the direction of the final repair construct were not parallel, an accessory portal was made to pull the tendon [11]. This suture loop was connected to the sterile hook of a digital tensiometer (DSV-50 N; IMADA Co., Ltd., Toyohashi, Japan), which could produce accurate measurements to two decimal points. In all cases, tension was forced to the tensiometer until the end of the tendon reached the lateral edge of the rotator cuff footprint, where the supraspinatus tendon was inserted anatomically. This complete footprint coverage was maintained for 20 s to achieve stress relaxation [28], and then the tension was recorded (Fig. 2). The tendon displacement at the anatomical insertion along this suture strand was also measured using a ruler. The stiffness of the musculotendinous unit was calculated using the following formula: stiffness = [(tension at anatomic insertion) / (displacement at anatomic insertion)], and was depicted by an almost straight line [29, 30]. Single-row repair or the knotless suture bridge technique was performed depending on the size of the tendon tear or the configuration of the tendon.

**Fig. 3:**
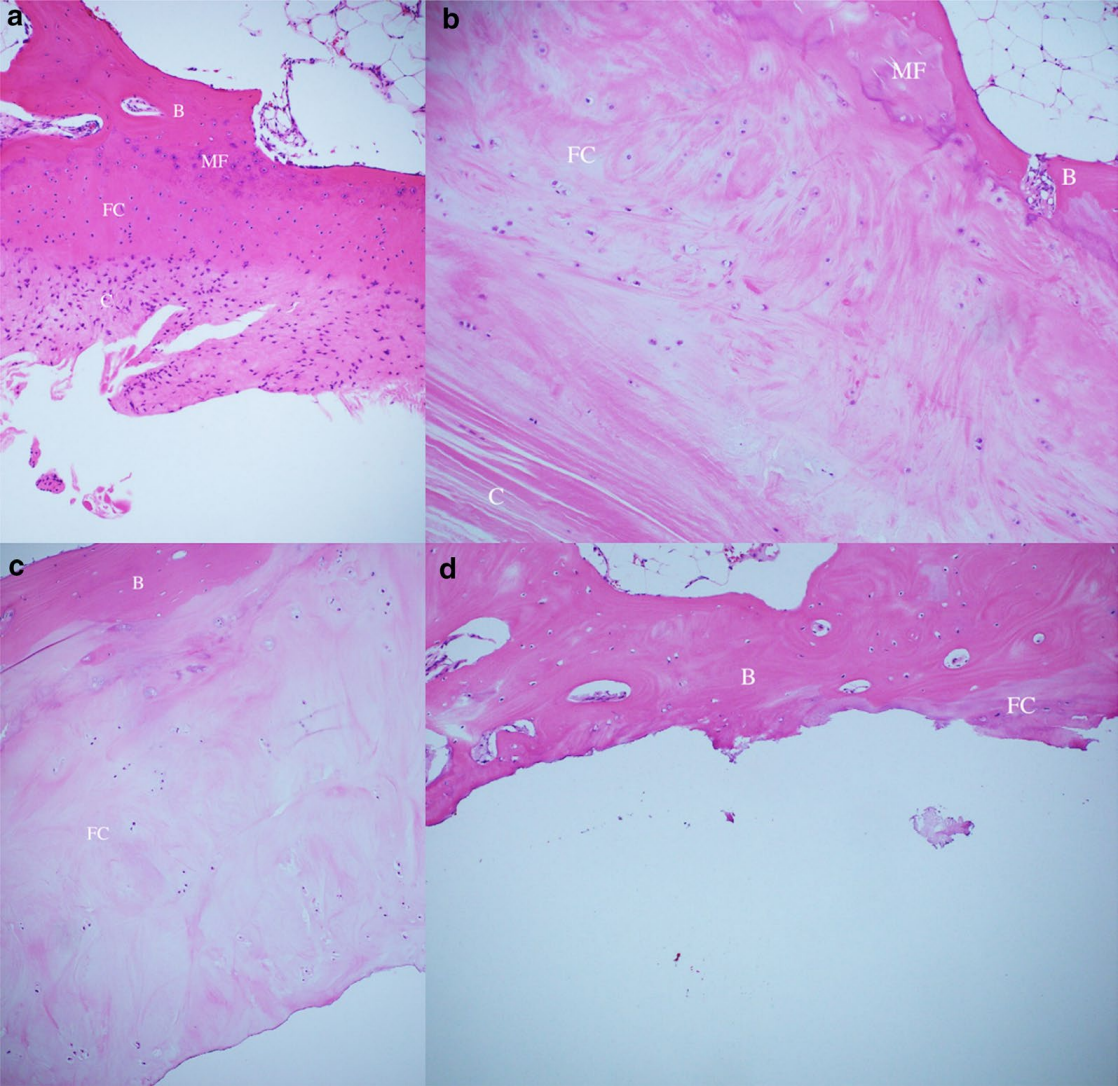
**a** Type I: Fissuring of the collagenous fiber layer with no abnormal changes in the other three layers. The coracoacromial ligament attachment at the undersurface of the acromion was composed of four layers: collagenous fiber (C), fibrocartilage (FC), mineralized fibrocartilage (MF), and bone (B) (× 100). **b** Type II: Reactive changes such as enlargement of the fibrocartilage layer. Irregularity of the tidemark was observed (× 100). **c** Type III: Disappearance of the collagenous fiber layer and remaining hypertrophic changes in the fibrocartilage layer (× 100). **d** Type IV: Disappearance of the fibrocartilage layer, resulting in exposure of the osseous layer (× 100)

The acromion and CAL specimens were fixed in 10% formalin and then cut into sections along the course of the CAL. After being dehydrated and embedded in paraffin, 4-μm-thick sections were cut from each block and stained with hematoxylin and eosin. Using an optical light microscope, the grade of CAL degeneration at the undersurface of the acromion was evaluated with reference to Takase and Yamamoto [31] (Fig. 3a–d). Slices were evaluated by an orthopedic surgeon in each institution in a blinded manner.

Of the 47 patients who were eligible for participation, 5 were excluded because of the inability to resect sufficient acromion and CAL tissue (*n* = 1), a partial-thickness RCT diagnosed by arthroscopy (*n* = 2), and incomplete footprint coverage due to a massive RCT (*n* = 2). The final study population comprised 19 patients with traumatic RCTs (Group T) and 23 patients with degenerative RCTs (Group D). The demographic data of the patients in both study groups are summarized in Table 1. Substantial demographic differences were not found between the two groups except that the prevalence of diabetes was significantly higher in Group D (*p* = 0.019). The duration of time from symptoms or trauma to surgery and preoperative fatty infiltration of the rotator cuff muscles was also comparable between the two groups. The type and degree of trauma in Group T are listed in Table 2.

**Table 1:** Summary of patients’ demographic and clinical data

Variable	Group T (*n* = 19)	Group D (*n* = 23)	*p* value
Age, years	72.5 ± 4.8	71.5 ± 4.8	0.493^a^
BMI, kg/m^2^	24.4 ± 2.9	24.1 ± 2.9	0.622^a^
Sex			
Male	9 (47.4)	16 (69.6)	
Female	10 (52.6)	7 (30.4)	0.145^b^
Diabetes	2 (10.5)	10 (43.5)	0.019^b^
Smoking	5 (26.3)	7 (30.4)	0.769^b^
Arm dominance	13 (68.4)	19 (82.6)	0.283^b^
Fatty infiltration			
SSC			
Grade 0	10 (52.6)	12 (52.2)	
Grade 1	9 (47.4)	9 (39.1)	
Grade 2	0 (0.0)	2 (8.7)	0.403^b^
SSP			
Grade 0	0 (0.0)	0 (0.0)	
Grade 1	4 (21.1)	7 (30.4)	
Grade 2	15 (78.9)	16 (69.6)	0.491^b^
ISP			
Grade 0	2 (10.5)	1 (4.4)	
Grade 1	12 (63.2)	11 (47.8)	
Grade 2	5 (26.3)	11 (47.8)	0.322^b^
TM			
Grade 0	13 (68.4)	16 (69.6)	
Grade 1	6 (31.6)	5 (21.7)	
Grade 2	0 (0.0)	2 (8.7)	0.361^b^
Duration from symptoms/trauma until surgery, days	114 (17–265)	138 (48–3600)	0.123^a^

Data are presented as mean ± standard deviation, *n* (%), or median (minimum–maximum)

Group T, traumatic rotator cuff tears; Group D, degenerative rotator cuff tears; BMI, body mass index; SSC, subscapularis; SSP, supraspinatus; ISP, infraspinatus; TM, teres minor

^a^Mann–Whitney *U*-test

^b^Chi square test

**Table 2:** Type and degree of trauma

Case	Type and degree of trauma
1	Falling from above height of shoulder
2	Falling onto outstretched arm from standing height
3	Falling while riding a motorcycle
4	Falling while riding a bicycle
5	Falling while riding a bicycle
6	Falling onto outstretched arm from standing height
7	Falling onto outstretched arm from standing height
8	Falling onto outstretched arm from standing height
9	Falling onto outstretched arm from standing height
10	Injured in high-speed motor vehicle accident
11	Falling onto outstretched arm from standing height
12	Falling onto outstretched arm from standing height
13	Falling onto outstretched arm from standing height
14	Injured in high-speed motor vehicle accident
15	Falling onto outstretched arm from standing height
16	Falling onto outstretched arm from standing height
17	Falling from greater than standing height
18	Falling onto outstretched arm from standing height
19	Falling from greater than standing height

### Statistical analysis

The sample size was calculated based on the stiffness of the musculotendinous unit from a previous study [30]. The mean stiffness of the musculotendinous unit in the complete footprint coverage group was 1.24 ± 0.42 N/mm. If the mean difference between two groups was 0.40 and allowing for a 0.42 standard deviation within groups, the power analysis showed that a sample size of 19 patients per group would have provided a statistical power of 80% with a two-sided level of 0.05 to detect significant differences. Statistical analyses were performed with IBM SPSS version 23 (IBM Corp., Armonk, NY, USA). Data are expressed as mean and standard deviation or median and minimum–maximum. The Mann–Whitney *U* test was used to define differences between two groups. The chi-square test was used to compare categorical variables. To investigate the strength of the relationship between the arthroscopic and histological evaluation of CAL degeneration, a Spearman rank correlation was performed between two raters for the classification systems described by Levy et al. [24] and Takase and Yamamoto [31]. The level of statistical significance was set at *p* < 0.05.

## Results

Arthroscopic findings are listed in Table 3. Although the mean size and displacement of the tendon tear were comparable between the two groups, the mean repair tension was significantly lower in Group T than in Group D (11.6 ± 5.3 vs. 23.6 ± 8.2 N, respectively; *p* < 0.001). The stiffness of the musculotendinous unit was also significantly lower in Group T than in Group D (0.56 ± 0.31 vs 1.09 ± 0.67 N/mm, respectively; *p* < 0.001). The proportion of patients with subscapularis tendon tear was similar between the two groups. The proportion of patients with LHB dislocation or subluxation was higher in Group T than in Group D (*p* = 0.014), although there were no differences in other pathological conditions of the LHB. The tendon status was significantly better in Group T than in Group D (*p* < 0.001). The arthroscopic grade of CAL degeneration (Copeland–Levy classification) was significantly milder in Group T than in Group D (*p* < 0.001). The histological grade of CAL degeneration (Takase classification) was also significantly milder in Group T than in Group D (*p* < 0.001) (Table 4). A statistically significant positive relationship was found between the Copeland–Levy classification and the Takase classification (r = 0.47, *p* = 0.002).

**Table 3:** Comparison of arthroscopic findings between the groups

Variable	Group T (*n* = 19)	Group D (*n* = 23)	*p* value
Tear size			
ML, mm	22.2 ± 6.6	24.4 ± 8.3	0.361^a^
AP, mm	22.0 ± 10.5	24.2 ± 9.7	0.245^a^
Medium	13 (68.4)	14 (60.9)	
Large	6 (31.6)	9 (39.1)	0.611^b^
Rotator cuff tendon status			
Good	13 (68.4)	0 (0.0)	
Moderate	6 (31.6)	15 (65.2)	
Poor	0 (0.0)	8 (34.8)	< 0.001^b^
Displacement, mm	21.7 ± 7.0	24.4 ± 8.3	0.273^a^
Tension, N	11.6 ± 5.3	23.6 ± 8.2	< 0.001^a^
Stiffness, N/mm	0.56 ± 0.31	1.09 ± 0.67	< 0.001^a^
LHB absent	0 (0.0)	3 (13.0)	0.102^b^
LHB dislocation or subluxation	7 (36.8)	1 (4.4)	0.014^b^
LHB tear			
Normal	7 (36.8)	11 (47.8)	
< 50%	9 (47.4)	8 (34.8)	
≥ 50%	3 (15.8)	4 (17.4)	0.699^b^
Subscapularis tendon tear	12 (63.2)	12 (52.2)	0.474^b^
Copeland–Levy classification			
Grade 0	4 (21.1)	0 (0.0)	
Grade 1	11 (57.9)	4 (17.4)	
Grade 2	4 (21.1)	13 (56.5)	
Grade 3	0 (0.0)	6 (26.1)	< 0.001^b^

Data are presented as mean ± standard deviation or *n* (%)

Group T, traumatic rotator cuff tears; Group D, degenerative rotator cuff tears; ML, medial-to-lateral; AP, anterior-to-posterior; LHB, long head of the biceps

^a^Mann–Whitney *U*-test

^b^Chi square test

**Table 4:** Comparison of histological grade of coracoacromial ligament degeneration between the groups

Takase classification	Group T (*n* = 19)	Group D (*n* = 23)	*p* value^a^
Type I	8 (42.1)	0 (0.0)	
Type II	10 (52.6)	9 (39.1)	
Type III	1 (5.3)	13 (56.5)	
Type IV	0 (0.0)	1 (4.4)	< 0.001

Data are presented as *n* (%)

Group T: traumatic rotator cuff tears, Group D: degenerative rotator cuff tears

^a^Chi square test

## Discussion

The most important findings of the present study were that the elasticity of the musculotendinous unit in traumatic RCTs was greater than in degenerative tears and the CAL degeneration in traumatic tears was milder than in degenerative tears. These results support our hypothesis that the preserved elasticity of the musculotendinous unit and mild CAL degeneration are specific features of traumatic RCTs in elderly patients.

Because of their prolonged life expectancy and improved medical treatment, many elderly patients can manage high functional demands in their daily life [32]. Arthroscopic repair of the rotator cuff allows elderly patients to obtain functional improvement similar to that of their younger peers [33, 34]. Although elderly patients are more likely to have degenerative changes [35], easier tendon reduction might improve repair integrity [36]. Our study clarified that the elasticity of the musculotendinous unit was preserved in traumatic RCTs and that it was possible to reduce the edge of the tendon to the footprint with less tension. Good tendon mobility might be advantageous for cuff healing in terms of avoiding excessive tension at the repair site [11–13], and information regarding the possibility of full footprint coverage with less tension would be useful for the surgeon to consider the unnecessity of an additional tension-free procedure preoperatively. Because the elasticity of the musculotendinous unit was preserved, most traumatic RCTs were less likely to require an additional procedure such as medialized repair [37], muscle advancement, and/or patch reinforcement [38]. One study showed that passive tension was increased in proportion to the tear size [28], and it is important to assess the distance required to reduce the edge of the tendon to the footprint. Our results demonstrated that the elasticity of the musculotendinous unit was better in traumatic than in degenerative RCTs despite the fact that the amount of preoperative fatty degeneration of the rotator cuff muscles and the size of the tear were comparable between the two groups. Therefore, our clinical results are consistent with previously published basic studies suggesting that the elasticity of the musculotendinous unit was preserved early after tendon detachment [8, 9].

The etiology of degenerative RCTs is multifactorial and has not yet been elucidated. The prevalence of diabetes was significantly higher in Group D than in Group T in our study. This finding is consistent with previous studies showing that diabetes was strongly associated with degenerative tears [39, 40]. Subacromial impingement is also believed to contribute to the development of RCTs [17]. Although degenerative changes in the coracoacromial arch are related to aging [41], Takase and Yamamoto [31] reported that histological changes in the undersurface of the acromion in patients without a cuff tear were merely minor changes compared with those in patients with a full-thickness cuff tear. Miyake et al. [42] reported that a larger size of the cuff tear was significantly associated with more severe damage to the acromion undersurface. In our study, both arthroscopic and histological CAL degeneration on the subsurface of the acromion was milder in patients with traumatic than degenerative RCTs, despite the fact that the size of the cuff tear was comparable between the groups. We also found a moderate positive correlation between the arthroscopic and histological grade of the CAL degeneration. Therefore, the arthroscopic evaluation of the CAL seemed to be helpful to distinguish traumatic RCTs from degenerative RCTs.

Namdari et al. [43] reported that patients with traumatic RCTs are more likely to have biceps tendon disorders such as subluxation or dislocation. The results of our study support these findings and demonstrate that biceps subluxation or dislocation is an important feature of traumatic RCTs. Preoperative diagnosis of biceps tendon disorders with imaging is challenging, and arthroscopic diagnosis seems to be the gold standard [44, 45]. Biceps tenotomy is usually chosen for elderly patients [46]; therefore, surgeons can explain possible complications, such as cosmetic deformity (Popeye sign), in patients with traumatic RCTs preoperatively.

Previous studies have shown that a thin and weak rotator cuff tendon is associated with retear of repaired rotator cuffs [36, 47]. In the current study, the status of the rotator cuff tendon was better in patients with traumatic than degenerative RCTs because the tendon was ruptured acutely due to trauma in the former. These results suggest that good tendon status is a characteristic of traumatic RCTs and might lead to better repair integrity.

Several limitations should be noted when interpreting our findings. First, some patients might have had asymptomatic degenerative RCTs prior to trauma because elderly individuals are more likely to have asymptomatic tears than younger individuals. Some of the patients’ tendons might have torn because of the progressive thinning of the rotator crescent due to aging-related degenerative changes [48]. The term “traumatic RCT” was strictly defined in our study, and patients with known RCT of the contralateral shoulder were excluded. Therefore, we believe that acute-on-chronic tears were unlikely among our patients. Second, we did not consider the histological findings of the tendon tissue. Although tendon histopathology is informative for understanding the etiology of the rotator cuff disease and helps to develop a novel investigation [49–51], the degree of histological tendinopathy is not strongly associated with patient demographics and tendon stiffness [15]. Third, we did not compare postoperative clinical outcomes and repair integrity between the two groups in this study. Fourth, we did not provide a definition of acute repair. The time from trauma to surgery was relatively long because most of the patients were introduced from other hospitals in our study. However, we believe that more chronic rotator cuff disease was excluded by including only patients with minimal fatty degeneration of the rotator cuff muscles. Fifth, the size of the traumatic RCTs in our study population was relatively small, although traumatic tears tended to be larger than degenerative tears [22]. We assume that the torn edge of the tendon was not severely retracted after trauma because elderly patients are more likely than younger patients to have lost the elasticity of the tendon [14, 15]. Sixth, degeneration of the CAL might be influenced by scapular movement. However, whether scapular dyskinesis is the cause or a consequence of degenerative rotator cuff pathology remains controversial [52]. Further research is needed to resolve these issues.

## Conclusion

Traumatic RCTs were characterized by preserved elasticity of the musculotendinous unit and milder CAL degeneration compared with degenerative RCTs even in elderly patients.

## Data Availability

The data sets used during the current study are available from the corresponding author on reasonable request.

## Abbreviations

RCT: Rotator cuff tear
CAL: Coracoacromial ligament
LHB: Long head of the biceps
MRI: Magnetic resonance imaging
